# P-1873. Infectious Disease Work Load Index: A Systematic Measurement of Patient Acuity and Overall Work Volume

**DOI:** 10.1093/ofid/ofaf695.2042

**Published:** 2026-01-11

**Authors:** Leah H Yoke, Pooja Bhattacharyya, Lisa So, Regina Lengermann, Cassandra Callas, Lauren Jatt, Emily A Rosen, Mabel Jimenez, Michael J Boeckh

**Affiliations:** University of Washington; Fred Hutch Cancer Research Center, Seattle, WA; Fred Hutch/University of Washington, Seattle, Washington; Fred Hutch/University of Washington, Seattle, Washington; FHCC, Seattle, Washington; Fred Hutch Cancer Center, Seattle, Washington; University of Washington, Seattle, Washington; Fred Hutchinson Cancer Center / University of Washington, Seattle, Washington; University of Washington, Seattle, Washington; Fred Hutchinson Cancer Center, Seattle, WA

## Abstract

**Background:**

Inpatient and outpatient Infectious Disease (ID) services are busy with medical decision making and care coordination. Measuring census alone does not account for patient acuity and complex care coordination, and these factors may not be fully captured in a traditional relative value unit (RVU). To better assess true work volume, a work load index (WLI) was created to measure census, care coordination tasks, and acuity to track effort on an ID oncology service and to help predict staffing needs.

**Figure 1:**
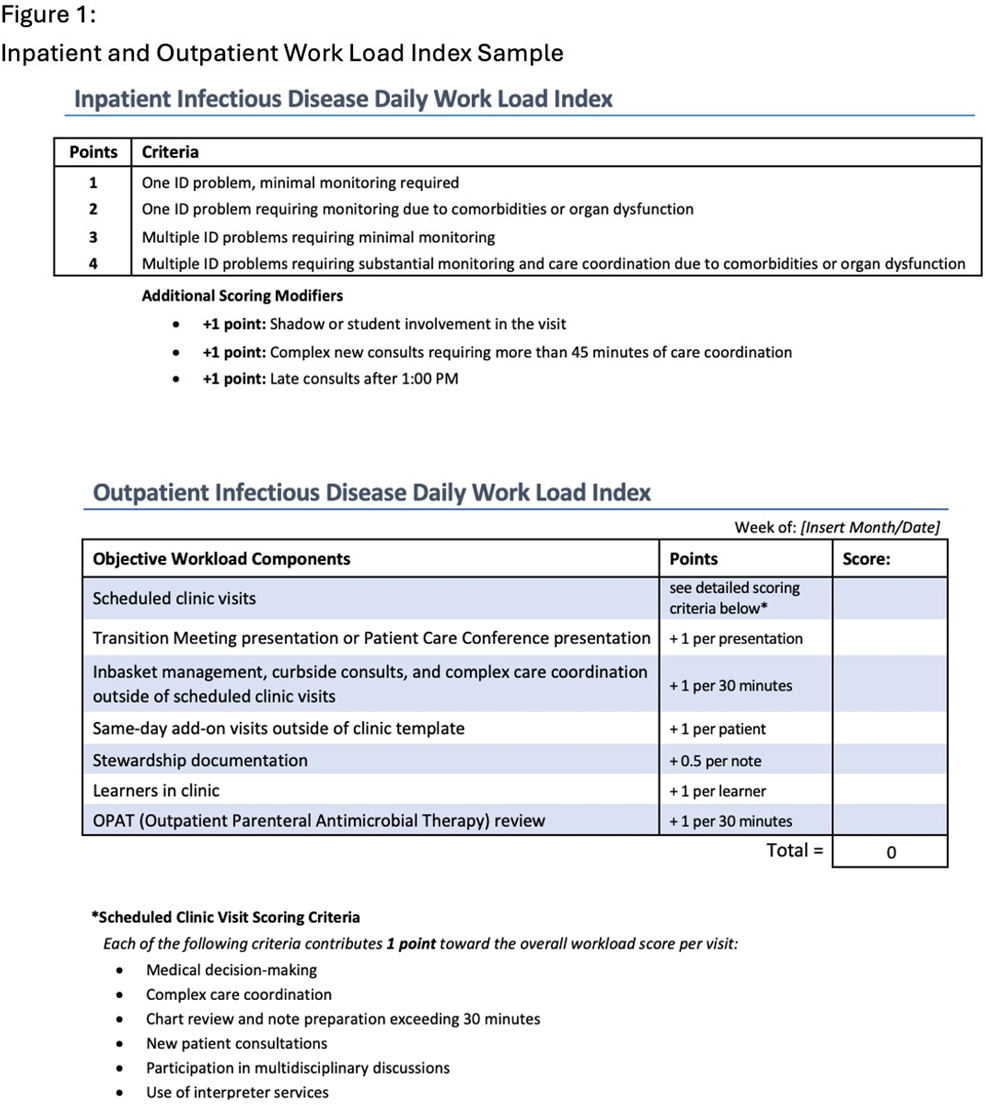
Inpatient and Outpatient Work Load Index Sample

**Figure 2:**
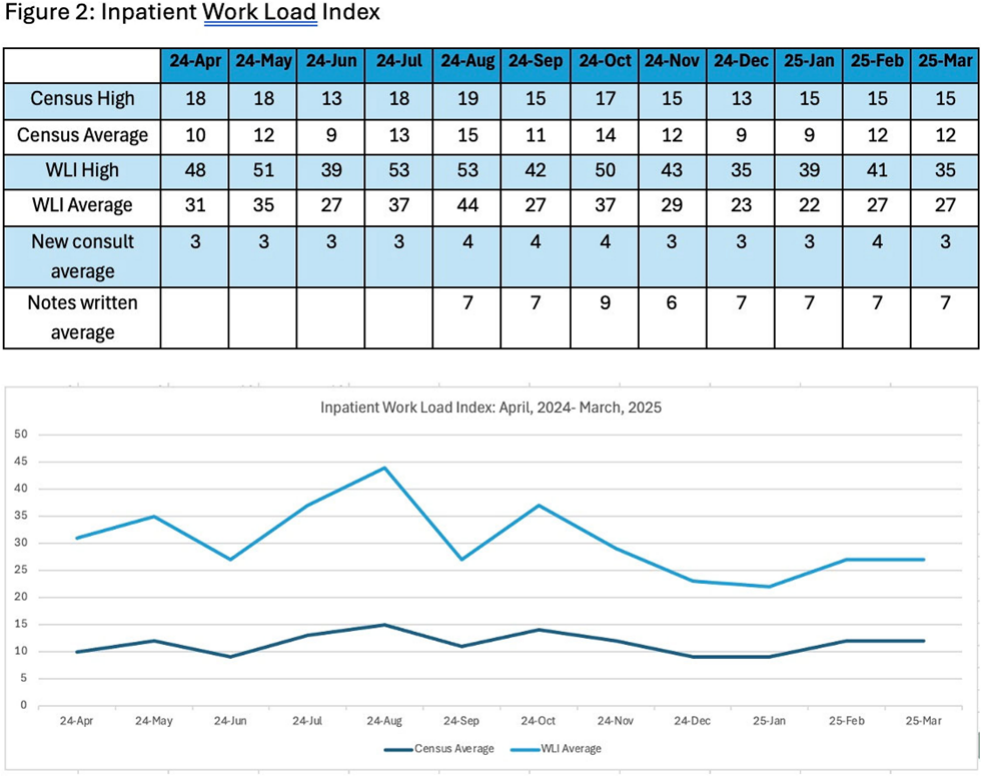
Inpatient Work Load Index

**Methods:**

An inpatient WLI was created in April 2024 to track daily number of consultations, acuity, and census (Figure 1). An outpatient WLI was created in October 2024 to track number and acuity of clinic visits, outpatient parenteral antimicrobial therapy monitoring, stewardship work, curbside consults, and non-billable patient care coordination (Figure 1). Data were submitted by team members daily.

**Figure 3:**
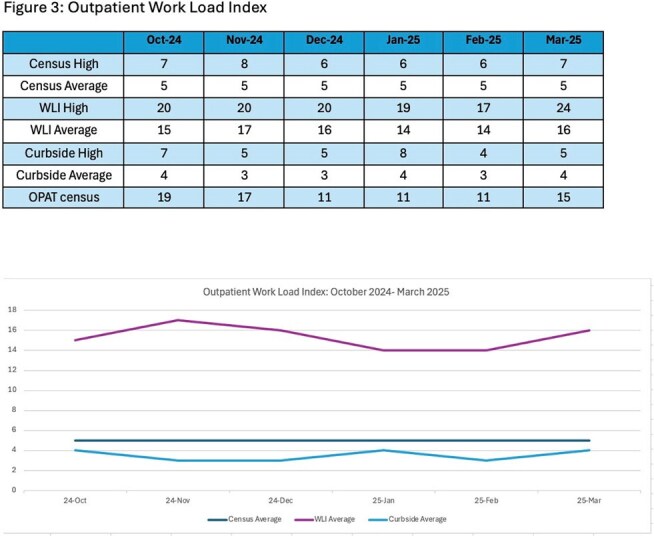
Outpatient Work Load Index

**Results:**

A WLI was enumerated from a combined score inclusive of care coordination and medical complexity in inpatient and outpatient settings. The inpatient WLI monthly daily average ranged from 22-44. Average daily notes written ranged from 6-9 with an average of 3-4 new consults per day (Figure 2). In the outpatient setting, daily census average by month was 5 patients, with WLI daily average ranging from 14-17 by month. Average curbside consultations ranged from 3-4 daily with 11-19 OPAT patients managed monthly (Figure 3).

**Conclusion:**

Because of the complexities of ID consultations, significant work may not be accounted for by tracking RVUs or census alone. We developed a WLI to quantify and track the complexity of medical decision making and care coordination provided by our consultative oncology service. While this allows for dynamic staffing in response to high WLI, it also may guide the need for resource allocation, including nursing, pharmacy, and provider positions, as well as optimization of billing. Future work includes multi-center validation and correlation of WLI with primary team census data to develop tools to predict times of increased work, thus allowing for staffing changes to optimize patient care.

**Disclosures:**

Michael J. Boeckh, MD PhD, Allovir: Advisor/Consultant|Ansun Biopharma: Grant/Research Support|AstraZeneka: Advisor/Consultant|AstraZeneka: Grant/Research Support|GSK: Grant/Research Support|Merck: Advisor/Consultant|Merck: Grant/Research Support|Moderna: Advisor/Consultant|Moderna: Grant/Research Support|Symbio: Advisor/Consultant|Vir Biotechnology: Grant/Research Support

